# Happy times, careers and happenstance in UK psychiatry: time, timeliness, timelessness, eternity and contemporality

**DOI:** 10.1192/bjb.2024.52

**Published:** 2024-08

**Authors:** George Ikkos, Nick Bouras

**Affiliations:** 1 Royal National Orthopaedic Hospital, London, UK; 2 Institute of Psychiatry, Psychology and Neuroscience, King's College London, London, UK

**Keywords:** History of psychiatry, psychiatric careers, neoliberalism and digitalisation, metacommunity psychiatry, politics and psychiatric professionalism

## Abstract

To address some challenges facing psychiatrists today we discuss issues of happenstance and fulfilment in psychiatric careers through some of the record and reflections of four psychiatrists since the 1950s. We trace the changes in psychiatry attendant to the transition from the welfare to the neoliberal state and=its contemporary postmodern culture. We highlight the crucial importance of political-cultural as well as technological developments in determining psychiatric service management and provision, and clinical practice and career outcomes. In the light of this impact, in a global era that some highly respected authorities consider in apocalyptic terms, we advocate for the incorporation of training in political awareness and activism in the psychiatric curriculum and practice. We suggest that this is necessary for social justice and patient welfare and that it will help safeguard psychiatric professionalism, conscience and self-esteem.

‘To be contemporary is to create one's time, not to reflect it. Or, to reflect it only not like a mirror but like a shield.’

Marina Tsvetaeva (p. 101)^1^

In a linked paper Poole^2^ invites us to reflect on career and happenstance in the life of Bertram Mandelbrote (1923–2010).^3^ Mandelbrote, a man of intelligence, integrity, initiative and intent, arrived as a Rhodes Scholar from South Africa in 1946 to study at the University of Oxford, where he researched Wilson's disease before training in psychiatry at the Maudsley Hospital in London. He was appointed Physician Superintendent at Coney Hill and Horton Hospitals in 1956, then Littlemore Hospital Oxford in 1959. Brittons ‘have never had it so good’ proclaimed in 1957 Prime Minister Harold McMillan (1894–1986). By retirement in 1988 Mandelbrote had pioneered locally the therapeutic community approach and overseen developments in psychiatric deinstitutionalisation and social rehabilitation, drug and alcohol rehabilitation, and new accessible counselling and psychotherapy services. A fulfilling record.

## Psychiatry 1950–1990: from the welfare to the neoliberal state

Martin Deahl, also a man of intelligence, integrity, initiative and intent, tells a different story, his own.^4^ He was appointed a consultant psychiatrist in 1990. Although this was in the vicinity of Tower Hamlets in East London, one of the poorest boroughs in the European Union at the time, i.e. different from Oxford, the two colleagues would have found things in common in their respective workplaces. Today, Deahl laments that training and services have fragmented, the imperatives of regulations have mandated spending hours before the computer rather than patients, continuity of care has been shattered and families burdened further. Time has gone by but has also fled from the consultation room. What separates Mandelbrote's and Deahl's career outcomes? Different times? Happenstance, in Poole's terms?

Mandelbrote was appointed Superintendent during the high tide of psychopharmacology in the 1950s and by the time he moved to work in Oxford the new liberal Mental Health Act 1959 had gained parliamentary ascent. He also had the ear of Enoch Powell (1912–1998), a highly controversial but able right-wing politician whose record as Minister of Health from 1960 to 1962 was better than most.^5^ A free marketeer, Powell's previous experience as junior minister at the Treasury enabled him to secure funding for the National Hospital Plan 1962, which created Britain's district general hospital (DGH) network. His libertarian views motivated advocacy for the closure of the crumbling and illiberal mental hospital network, savings from which would fund the DGHs. Consequently, psychiatric patients would now be treated in the community and DGHs. These were the services that Deahl and contemporary consultants were appointed into.

The working lives of Mandelbrote and Deahl share the decade of the 1980s, a period of great social strife in Britain, a key aspect of which was documented in the recent Channel 4 TV series ‘The Miners’ Strike 1984: The Battle for Britain’ (2024). Its outcome changed Britain decisively. Mandelbrote was appointed Superintendent during the post-war Welfare State consensus but the significance of the 1980s is that by 1990 Britain had become neoliberal. Things took a fateful turn with the National Health Service and Community Care Act 1990, surely a deliberate misnomer by a Conservative government (1979–1990) led by Margaret Thatcher (1925–2013), who was viscerally hostile to the NHS and had infamously been quoted as saying ‘there is no such thing as society’. Dr Mandelbrote's appointment was ‘timely’, Dr Deahl's not, if we may put it this way.

Psychiatric services do some good but by the time we finished editing *Mind, State and Society*^6^ we had a strong sense that they were better set up to meet the needs of venture capital than patients. The Swinging Sixties notwithstanding, with the empire gone and high consumer consumption, Britain has struggled since that decade with balance of payments deficits and debt, which became worse with the 1973 oil crisis. The importance of neoliberalism as a response to such challenges includes that it significantly handed over national governance and control to international finance.^7^ It is a timeless truth that he who pays the piper calls the tune. In former Prime Minister Theresa May's memorable phrase, this now became the ‘citizens of nowhere’. Their priority has never been the welfare of the most vulnerable but the minimum possible welfare provision to maintain the efficiency and legitimacy of earnings from capital investment.^8^

## Psychiatry 1990–2020: neoliberalism and the postmodern condition

As contemporary of Poole and Deahl, G.I. can add his reminiscences to theirs. He was invited in 2007 to become Medical Director of his mental health trust. Although he had been Chair of the Royal College of Psychiatrists’ London Division for 5 years, where he had actively advocated psychiatrists’ engagement in medical management, and was continuing as its Honorary Treasurer, he was shocked when he met the trust's Interim Chief Executive, who presented a strategic plan concocted by one of the big corporate consulting companies. It had nothing to do with how he had been trained, nor with what made professional sense and, after much agonising, withdrew his application. It was a grave error of the New Labour government (1997–2010), an act of political-administrative vandalism, that although they invested in services, securing a 45% increase in consultant psychiatrist numbers between 1999 and 2004,^9^ they turned to American business management practices. Rather than build organically as Poole summarises, they developed governance systems that favoured financial (ac)counting over clinical accountability.^10^

Beware oversimplification! The 1960s–1970s were not ‘happy times’^11–13^ and there have been some improvements since.^14,15^ Had psychiatry matched the impressive biomedical advances of other medical specialties, often based on technology, it might have turned out different, but this was always improbable. Additionally, declining local fertility rates, growing inbound migration and increasing longevity have changed the composition and nature of our communities. Furthermore, computing and digitalisation brought about ‘neo-communities’^16^ and other massive unforeseen changes. We have argued elsewhere that commencing in the 1990s they seeded the beginnings of a new era beyond community psychiatry, the era of metacommunity psychiatry.^17^ In Greek, meta means after, i.e. after community. One can debate when it started but now, post COVID-19 and in the Age of AI,^18^ it has arrived. All that Deahl laments is a key aspect of it and truly it is an eternity away from Mandelbrote's era.

Mandelbrote carried the aura and authority of the Physician Superintendent over to the community, perhaps its menace too. These have expired with the phantasmagoria of financial capitalism, globalisation, technology, big data, and commodification of knowledge and health, i.e. the whole panoply of postmodernism. Perhaps had more psychiatrists read when it was first published in 1979 Jean Francois Lyotard's admirably lucid *La Condition Postmoderne: Rapport sur le Savoir*’,^19^ we might have been better prepared. Key themes included numbers dominating over narratives, and the commodification of knowledge, corporatisation of health services and demoralisation of the professions. And had we withstood Fredric Jameson's exuberantly verbose *Postmodernism: Or, the Cultural Logic of Late Capitalism*^20^ we might have anticipated postmodernism's ‘attenuation of affect’ and the bureaucracy associated with it. At its grotesque extreme, this would demand that psychologists and psychiatrists become the administrators of misery and madness in the neoliberal state rather than enlightened professional healers.

## Psychiatry today: metacommunity and politics

Ultimately, we must face our contemporality. Those will fare best and contribute most who grasp and address current realities: evidence, modern management techniques, big data, understanding influence, teaching: all this firmly tethered to clinical ethics and wisdom. Beyond ‘leadership’, we must also grasp the grand Politics, which glaringly determines our practice, in order both to be prepared for it and speak out about today's political economy, which increases rates of common and other mental health problems,^21^ dehumanises services and delivers second-best services to those with more severe conditions.^22^ To voice concerns effectively in the public sphere, locally and beyond, this broader political know-how about ideology and power, including how they affect health and public administration, needs to be part of both our professional formation and continuing development. We are not entirely at the mercy of happenstance but have a part in shaping it. Our tough and more complex times notwithstanding, Mandelbrote's story suggests that much can be achieved with low tones, clear thinking, presence, a can-do attitude, loyalty and the informal knack for engaging and trusting galvanised colleagues. For these to happen, however, we must be effectively supported by the state and civil society.^23^ After all, psychiatry is a profession, even a vocation, but not a sacrifice.

Poole's and Deahl's papers are about the soul of the profession.^2,4^ But let's not beat about the bush. The difficulties we face are not unique but shared. As distinguished American political scientist Wendy Brown observed in her 2019 Tanner Lectures on Human Values, we urge younger people to ‘Build your resumé, cultivate your networks, find your mate … but also, save for an unaffordable home and unlikely retirement, plan for the end of democracy, and an uninhabitable planet. Most young people are in a mode of pre-apocalyptic survivalism, as are we all to some extent’ (p. 104).^24^ This reality has political, epidemiological and daily clinical practice implications for psychiatrists, all equally crucial: at mega-, macro- and microlevels. Fulfilment depends on happenstance, but our times, conscience and self-esteem also depend on our response to it.

## Data Availability

Data availability is not applicable to this article as no new data were created or analysed in this study.

## About the authors

**George Ikkos** is a consultant psychiatrist in the Department of Liaison Psychiatry at the Royal National Orthopaedic Hospital, London, and immediate past Chair of the Royal College of Psychiatrists’ History of Psychiatry Special Interest Group (HoPSIG), London, UK. **Nick Bouras** is Emeritus Professor of Psychiatry at the Institute of Psychiatry, Psychology and Neuroscience, King's College London, UK. The present paper is the fifth in an ongoing dialogue and collaboration between the two authors that was commenced almost 10 years ago to address issues relating to N.B.'s concept of ‘metacommunity psychiatry and mental health’ and the history of psychiatry, and includes joint editorship of *Mind, State and Society: Social History of Psychiatry and Mental Health in Britain 1960–2010*, which was runner up in the Association of American Publishers’ PROSE 2022 Awards in the section History of Science, Medicine and Technology (available open access from https://doi.org/10.1017/9781911623793).
